# Neural regulation of bone: from central neural circuits to peripheral innervation of the skeletal stem cell niche

**DOI:** 10.1038/s41413-026-00534-4

**Published:** 2026-04-21

**Authors:** Zihan Chen, Zhengqiong Luo, Matthew B. Greenblatt, Zuoxing Wu, Ren Xu

**Affiliations:** 1 Collaborative Innovation Centre of Regenerative Medicine and Medical BioResource Development and Application Co-constructed by the Province and Ministry, Guangxi Medical University, Nanning, Guangxi China; 2 The First Affiliated Hospital of Xiamen University-ICMRS Collaborating Center for Skeletal Stem Cells, Xiamen Cell Therapy Research Center, The First Affiliated Hospital of Xiamen University, School of Medicine, Faculty of Medicine and Life Sciences, Xiamen University, Xiamen, Fujian China; 3 Xiamen Key Laboratory of Regeneration Medicine, Fujian Provincial Key Laboratory of Organ and Tissue Regeneration, School of Medicine, Xiamen University, Xiamen, Fujian China; 4 Department of Pathology and Laboratory Medicine, Weill Cornell Medical College, New York, NY USA; 5 Skeletal Health and Orthopedic Research Program, Hospital for Special Surgery, New York, NY USA

**Keywords:** Bone, Diseases

## Abstract

The nervous system has emerged as a multi-scale regulator of bone biology, integrating central neural circuits with peripheral innervation to control skeletal homeostasis and repair. While bone remodeling is classically described as being governed by coupling between bone formation and resorption, neural signaling provides an additional hierarchical layer that links organism-level cues to local skeletal stem/progenitor cell niches. This review presents a mechanistic framework for the neuro–bone regulatory network across three hierarchical levels. First, we examine central regulation, in which hypothalamic circuits integrate hormonal and metabolic signals via circumventricular organs to modulate endocrine outputs such as parathyroid hormone (PTH), thereby establishing circadian rhythms and systemic control of bone metabolism. Second, we analyze peripheral neural communication, where sensory inputs triggered by injury or inflammation, along with autonomic efferent signaling, including β-adrenergic pathways, directly influence osteolineage and stromal cells. These signals recalibrate cellular metabolic states, differentiation programs, and regenerative responses, linking pain perception with tissue repair mechanisms. Third, we investigate the bone marrow niche, where distinct subtypes of nerve fibers release a diverse array of neurotransmitters and or neuromodulators that shape the microenvironment of skeletal stem and progenitor cells (SSPCs) as well as downstream osteoprogenitors, thereby regulating proliferation, lineage commitment, and quiescence. Collectively, these findings delineate an integrated model of neural regulation of bone spanning central, peripheral, and local niche levels, providing a foundation for testable hypotheses in neuro-osteobiology.

## Introduction

Bone mass is maintained by the dynamic coupling of osteoclast-mediated resorption and osteoblast-mediated formation. Disruption of this coupling drives osteoporosis, fragility fractures, and tumor-associated osteolysis, imposing substantial health and economic burdens.^1–4^ Interventions that target only osteoclasts or osteoblasts often yield incomplete or transient benefit, implying that higher-order regulatory systems gate skeletal homeostasis and regeneration.^5–7^

Converging evidence now positions the nervous system as an upstream controller of bone remodeling. The central nervous system (CNS) integrates hormonal and metabolic inputs and, through autonomic and sensory output, sets the metabolic tone of the bone-marrow niche—thereby setting thresholds for bone maintenance and repair.^8–10^ Advances in genetic and imaging technologies have transformed the “neuro–bone axis” from a correlative concept into a testable framework, demonstrating a causal effect of neural influence on skeletal cells.^11–13^

Previous studies have demonstrated that, within the CNS, the hypothalamus acts as a hub for internal milieu control. Nuclei such as the arcuate nucleus (ARC) and PVN integrate signals conveyed by circumventricular organs (CVOs)—including the median eminence (ME), subfornical organ (SFO), organum vasculosum of the lamina terminalis (OVLT), area postrema (AP), and subcommissural organ (SCO)—which partially bypass the blood–brain barrier. Fenestrated capillaries and specialized glial contacts at the ME/ARC interface facilitate endocrine-to-neural transduction with high sensitivity.^14^ Recent work indicates that parathyroid hormone-sensing gamma-aminobutyric acid-releasing (GABAergic) neurons in the SFO centrally regulate PTH and bone metabolism via a downstream pathway involving the PVN and sympathetic nervous system.^15^ Likewise, leptin-responsive hypothalamic pathways modulate bone remodeling, supporting an integrated “central–humoral–bone” model.^16^

Classical peripheral reflex arcs mediate rapid, bidirectional communication between the nervous system and skeletal tissues. Sensory fibers richly innervate bone and marrow and detect injury and inflammation. Prostaglandin E2 (PGE2) acting on prostaglandin receptor 4 (EP4) activates peripheral sensory neurons, and ascending activity induces cAMP/CREB-dependent plasticity in the CNS, coupling nociception to regenerative set-points.^17–22^ Descending commands and autonomic outputs—particularly noradrenergic/β-adrenergic signaling—act directly on osteoblasts and stromal cells to reprogram metabolism and lineage decisions, forming a closed sensory–central–autonomic loop that shapes skeletal remodeling.^23^

While prior research has predominantly examined classical neural pathways in bone regulation, recent findings highlight a highly integrated intraosseous neurovascular network. Within this network, both nerve fibers and vascular endothelial cells modulate the local skeletal niche through the release of specific signaling molecules. Within this niche resides a discrete population of skeletal stem cells (SSCs) that sustains osteoblast output and stromal renewal in mouse and human bone.^24–34^ Endothelial angiocrine factors together with neuronal transmitters and neuropeptides orchestrate the proliferation and lineage commitment of SSCs and downstream osteoprogenitors, while immune cells (e.g., macrophages and dendritic cells) sculpt the niche through cytokines and metabolic substrates such as lactate and glutamate, as well as micronutrient handling (iron, manganese, zinc).^35–40^ This multicellular interaction unit explains why neural modulation often coincides with angiogenesis, immune polarization, and bone remodeling, and it underpins the therapeutic appeal of targeting Type-H vessels that couple angiogenesis to osteogenesis.

This review synthesizes the crosstalk between the nervous and skeletal systems and establishes neuromodulation as a key determinant of bone function. We first delineate central (hypothalamic) control, then peripheral sensory and autonomic pathways, and finally the dual—anabolic versus catabolic—effects of neural innervation within the bone marrow niche. Overall, we have delineated and summarized the classical mechanisms of neural regulation in bone remodeling, encompassing both central and peripheral pathways, with a particular focus on the innervation of the skeletal stem cell microenvironment. These advances will inform future neuroregulatory strategies aimed at treating skeletal diseases, thereby paving the way for more effective therapeutic interventions.

## Classical central nervous system regulation of bone

**Fig. 1:**
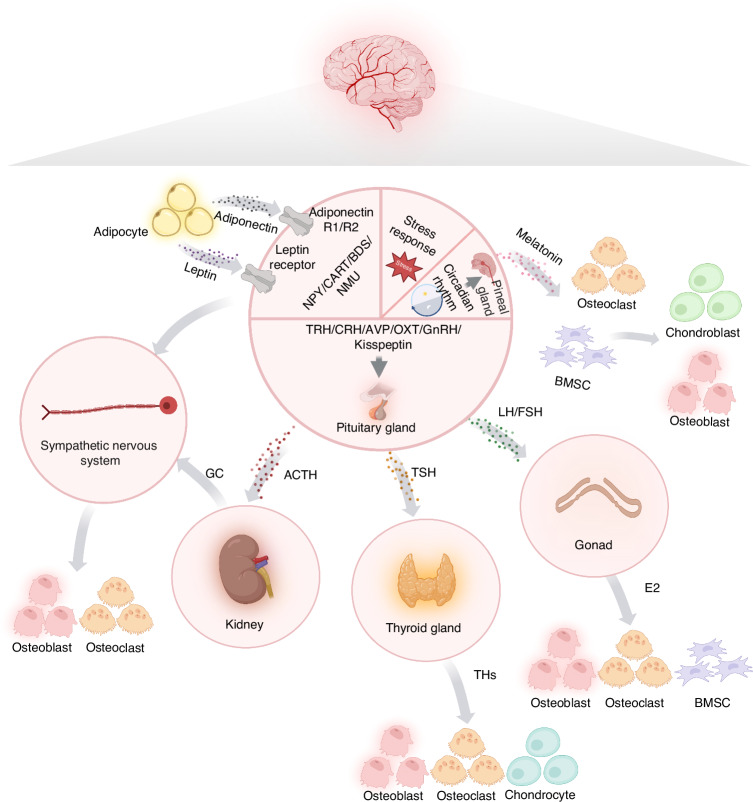
The key regulatory axis employed by the classical central nervous system for bone regulation. The regulation of bone metabolism by the central nervous system involves multiple axes, including the sympathetic nervous system axis, the hypothalamic-pituitary axis, and the circadian rhythm axis. Distinct populations of neurons within the central nervous system participate in this regulatory network, working in concert to maintain bone homeostasis. (Created with BioRender.com.) ACTH adrenocorticotropic hormone, AVP arginine vasopressin, BDS brainstem-derived serotonin, BMSC bone marrow-derived mesenchymal stem cell, CART cocaine-and amphetamine-regulated transcript, CRH corticotropin-releasing hormone, E2 estradiol, FSH follicle-stimulating hormone, GC glucocorticoid, GnRH gonadotropin-releasing hormone, LH luteinizing hormone, NMU neuromedin U, NPY neuropeptide Y, OXT oxytocin, THs thyroid hormones, TRH thyrotropin-releasing hormone, TSH thyroid-stimulating hormone

Recent studies have revealed that specific neuronal populations in the brain exert long-range control over bone metabolism. Hypothalamic nuclei such as the ARC, PVN, and ventromedial hypothalamus (VMH) integrate hormonal, metabolic, and inflammatory signals and relay them through autonomic and neuroendocrine outputs (Table 1). These central circuits maintain homeostasis, couple bone repair with systemic energy balance, and orchestrate circadian rhythms of bone remodeling.^41^ In this section, we summarize four regulatory axes—sympathetic, parasympathetic, hypothalamic–pituitary, and circadian rhythms—that collectively define the neural framework governing skeletal homeostasis (Fig. 1).

**Table 1:** Key factors of the central nervous system on the main regulatory axis of bone

Factors	Sources	Receptors	Axis	Effects	References
Leptin	Peripheral adipocytes	Lepr	Sympathetic axis	Sympathetic nerve stimulation exerts a dual regulatory effect on bone metabolism. On one hand, it acts through the Adrb2 receptor on osteoblasts to promote bone resorption by mediating RANKL expression. On the other hand, it suppresses osteoblast proliferation and leads to reduced bone mass by modulating the expression of c-Myc and Cyclin D through the molecular clock mechanism.	^51,174^
Adiponectin	Peripheral adipocytes	AdipoR1/ R2	Sympathetic axis	Intracerebroventricular (ICV) infusion of adiponectin reduced sympathetic tone, enhanced osteoblast differentiation, suppressed osteoclast differentiation and RANKL expression, promoted osteogenic differentiation of bone marrow stromal cells (BMSCs), and increased bone mass accumulation.	^53^
BDS	Brainstem	VMH Htr2c	Sympathetic axis	BDS reduces sympathetic nervous system activity, thereby promoting bone mass accumulation.	^54,274^
NPY	NPY expression in ARC	Y2 receptor expression in ARC	Remain to be determined	ICV infusion of NPY in mice leads to a decrease in bone mass. In contrast, genetic deficiency of *Npy* conversely causes a significant increase in bone mass. This phenotype is also observed in mice lacking the Y2 receptor, indicating the critical role of the NPY-Y2 signaling pathway in bone homeostasis.	^16,55,56^
		Y6 receptor expression in SCN	Circadian Rhythm Axis	The pathway by which NPY signals through the Y6 receptor in the suprachiasmatic nucleus inhibits bone resorption while simultaneously stimulating bone formation.	^99,100^
CART	Hypothalamus	Remain to be determined	Remain to be determined	CART directly inhibits RANKL signal transduction and subsequent bone resorption through multiple steps.	^52,275^
NMU	The dorsal medial nucleus of the hypothalamus	NMUR2	Remain to be determined	NMU modulates bone formation through the leptin-SNS-Adrb2 axis via central relay and unidentified pathways. Additionally, mice deficient in NMU exhibit a high bone mass phenotype.	^105^
SCT	VMH	SCTR	Sympathetic axis	Ablation of VMH-derived SCT or its receptor (SCTR) markedly enhances sympathetic outflow, leading to substantial bone loss. Conversely, VMH-specific SCT overexpression ameliorates sympathetic tone and promotes bone mass accrual.	^276^
SIRT1	Brainstem	Tph2, MAO-A transcription regulation	Sympathetic axis	SIRT1 reduces bone mass by promoting serotonin-mediated upregulation of brain catecholamine synthesis, thereby enhancing SNS signaling.	^277^
FoxO1	LC/ glomeruli of sympathetic ganglia	DBH transcription regulation	Sympathetic axis	FoxO1 modulates dopamine DBH expression in the locus coeruleus, thereby regulating catecholamine synthesis and affecting bone metabolism.	^278^
IL-1	Glia and neuron cells	IL-1RI	Central IL-1-PSNS-bone axis	Central IL-1 signaling acts to promote bone mass accrual by suppressing bone resorption.	^58,61^
GC	Renicapsule	GR	HPA	GCs decrease the number and function of osteoblasts, promote osteoblast apoptosis, and enhance the activity and lifespan of osteoclasts. These actions lead to reduced bone mass and ultimately result in GIO. Additionally, GCs indirectly affect bone metabolism by modulating the levels of hormones such as PTH, GH, and estrogen.	^66,67^
CCN3	KISS1 neuron in ARC	Remain to be determined	HPG	CCN3 directly stimulates the activity of mouse and human skeletal stem cells in a sex hormone-independent manner, enhancing bone remodeling and accelerating fracture repair in mice.	^76^
FSH	Hypophysis	FSHR	HPG	FSH directly stimulates bone resorption via its receptor on osteoclasts.	^279^
Estrogen	Oarium	ER	HPG	Estrogen exerts a dual regulatory effect on bone remodeling: it promotes osteoblast differentiation from mesenchymal stem cells (MSCs) while inhibiting osteoblast apoptosis, thereby extending osteoblast lifespan and enhancing bone formation. Concurrently, estrogen suppresses osteoclast formation and induces osteoclast apoptosis, which serves to limit bone resorption.	^280–282^
Androgen	Orchis	AR	HPG	Hypogonadism represents a leading etiological factor for osteoporosis in males.	^283^
TSH	Hypophysis	TSHR	HPT	It modulates the functions of osteoblasts and osteoclasts through multiple mechanisms.	^284–286^
THs	Thyroid gland	TRα1/TRβ1	HPT	It modulates the functions of osteoblasts, osteoclasts, and chondrocytes through multiple mechanisms, thereby coordinating bone development.	^78–81,287–290^
OXT	Hypophysis	Oxtr	Other Hypothalamic Outputs	It up-regulates BMP-2, stimulates osteoblast mineralization, and promotes the expression of Schnurri-2/3, Osterix, and ATF-4. In addition, it exerts dual effects on osteoclasts.	^83^
AVP	Hypophysis	Avpr1α/ Avpr2	Other Hypothalamic Outputs	The injection of AVP in wild-type mice resulted in increased bone resorption by osteoclasts and decreased bone formation by osteoblasts.	^84,85^
Melatonin	Epiphysis	MT1/ MT2	Circadian Rhythm Axis	It promotes osteogenesis and vascularization during bone repair, while inhibiting osteoclast generation and driving the differentiation of BMSCs into osteoblasts and chondroblasts.	^291^

*Adrb2 β2*-adrenergic receptors, *ARC* arcuate nucleus, *AVP* arginine vasopressin, *BDS* brainstem-derived serotonin, *CART* cocaine-and amphetamine-regulated transcript, *CCN3* cellular communication network factor type 3, *DBH* dopamine beta-hydroxylase, *FSH* follicle-stimulating hormone, *GC* glucocorticoid, *GH* growth hormone, *GIO* glucocorticoid-induced osteoporosis, *HPA* hypothalamic-pituitary-adrenal, *HPG* hypothalamic-pituitary-gonadal, *HPT* hypothalamic-pituitary-thyroid, *ICV* intracerebroventricular, *IL-1* interleukin-1, *Lepr* leptin receptors, *MAO-A* monoamine oxidase A, *MSCs* mesenchymal stem cells, *NMU* neuromedin U, *NPY* neuropeptide Y. *OXT* oxytocin, *PSNS* parasympathetic nervous system, *PTH* parathyroid hormone, *SCT* secretin, *SIRT1* sirtuin 1, *SNS* sympathetic nervous system, *THs* thyroid hormones, *TSH* thyroid-stimulating hormone, *VMH* ventromedial hypothalamus

### Central sympathetic axis

Having outlined how sympathetic outputs shape skeletal homeostasis, we next delineate the central circuitry that gives rise to these signals. Retrograde tracing with PRV152 revealed that the virus injected into the femur initially transduced sympathetic nervous system (SNS) postganglionic neurons at the L1-L2 level. This subsequently labeled SNS preganglionic neurons in the intermediolateral column (IML) of the lower thoracic spinal cord, which in turn projected to superior central nuclei, including the hypothalamus and brainstem.^42,43^ These findings establish a CNS–SNS regulatory loop in which specific hypothalamic nuclei receive afferent metabolic and inflammatory signals and send descending outputs through the PVN, rostral ventrolateral medulla (RVLM), and IML, thereby modulating sympathetic tone to bone.^44^ The excitability of presympathetic neurons is driven by glutamate and other excitatory neurotransmitters, which activate N-methyl-D-aspartate (NMDA) and α-Amino-3-hydroxy-5-methyl-4-isoxazolepropionic acid (AMPA) receptors in the PVN and RVLM circuits. This process is subject to stringent regulation by calcium/calmodulin-dependent kinase II (CaMKII), which controls NMDA receptor activity and phosphorylation levels.^45–49^

Bone mass is modulated by central sympathetic outflow, which regulates osteoblast function. This sympathetic excitability is dependent on circulating leptin, derived from adipocytes.^50^ Mechanistically, leptin acts on leptin receptors (LepR) in the hypothalamus, enhancing sympathetic output and thereby reducing bone mass. Subsequent sympathetic activation inhibits osteoblast proliferation via the β2-adrenergic receptor (Adrb2) and its downstream PKA-ATF4 signaling pathway, while concurrently promoting osteoclastogenesis by increasing Rankl(receptor activator of nuclear factor-κB ligand) expression. In contrast, the suppression of sympathetic activity promotes bone formation.^51,52^ Conversely, adiponectin (APN), another adipocyte-derived hormone, exerts opposite effects, reducing sympathetic activity, restraining osteoclast activation, and promoting osteoblast differentiation.^53^

Neuropeptides provide an additional regulatory layer. Brain-derived serotonin (BDS), cocaine- and amphetamine-regulated transcript (CART), neuropeptide Y (NPY), and neuromedin U (NMU) are all involved in linking sympathetic tone to bone remodeling. For instance, BDS originating from the brainstem binds to Htr2c receptors on VMH neurons, triggering the activation of calcium/calmodulin-dependent protein kinase kinase β (CaMKKβ). This, in turn, phosphorylates and activates calcium/calmodulin-dependent protein kinase IV (CaMKIV), which subsequently leads to the phosphorylation of cAMP response element-binding protein (CREB) at Serine 133. This signaling cascade reduces peripheral sympathetic activity, thereby promoting bone mass accumulation.^54^ In contrast to its divergent roles in appetite and energy balance, NPY signaling in the ARC functions as a negative regulator of bone mass, a role consistent with that of leptin in bone metabolism.^16,55,56^

Collectively, these studies demonstrate that the central nervous system integrates multiple signals—including metabolic cues, adipokines, and neuropeptides—to control sympathetic output. The sympathetic nervous system subsequently exerts a negative influence on bone mass by both suppressing osteoblast proliferation directly and enhancing osteoclast-driven bone resorption indirectly.

### Central parasympathetic axis

Building on the characterization of sympathetic circuits, we proceed to the parasympathetic counterpart that provides balance. Experimental tracing with pseudorabies virus has demonstrated that parasympathetic nervous system (PSNS) signals project to the spinal cord and higher brainstem nuclei. Choline acetyltransferase (ChAT)-positive vagal efferents innervating bone have been identified, and vagus nerve stimulation (VNS) significantly increases bone mineral density in rodents. Conversely, disruption of parasympathetic activity reduces bone mass, underscoring the functional importance of this axis.^57–60^

Compared to the sympathetic nervous system, current understanding of the CNS mechanisms that regulate parasympathetic outflow and directly influence bone homeostasis remains relatively limited. It is generally accepted that the CNS mediates PSNS-regulated bone homeostasis through two primary pathways. Central IL-1 signaling modulates bone metabolism through PSNS-derived nicotinic cholinergic fibers, as evidenced by significant bone loss following either vagotomy or α7nAChR deletion in mice.^58,61^ Furthermore, brainstem Chrm3 expression and activity influence bone remodeling by inhibiting sympathetic tone.^62^

Collectively, these results indicate that the central parasympathetic axis plays an essential role in maintaining normal bone mass by antagonizing the sympathetic axis. However, the underlying mechanisms remain incompletely understood. Further research is needed to elucidate how the central nervous system regulates parasympathetic outflow and directly participates in the modulation of bone homeostasis.

### Hypothalamic-pituitary axes

The hypothalamus acts as a central hub that integrates hormonal, metabolic, and neuronal signals from peripheral tissues and transduces them into endocrine outputs. These outputs operate through several hypothalamic–pituitary axes, each exerting distinct but interconnected influences on bone metabolism. By coordinating stress responses, reproductive function, and thyroid activity, the hypothalamus establishes systemic frameworks that link whole-body physiology to skeletal homeostasis.^63,64^

#### Hypothalamic-pituitary-adrenal (HPA) axis

The HPA axis is a central regulator of stress responses. Corticotropin-releasing hormone (CRH) and arginine vasopressin (AVP) released from the PVN stimulate adrenocorticotropic hormone (ACTH) secretion, leading to glucocorticoid (GC) production. While physiologic GCs maintain energy balance and bone turnover, chronic GC elevation—as observed in stress, Cushing’s syndrome, or prolonged steroid therapy—suppresses osteoblast proliferation, enhances osteoclastogenesis, and induces osteoporosis.^65–67^

#### Hypothalamic-pituitary-gonadal (HPG) axis

The HPG axis regulates reproductive hormones that also strongly influence skeletal metabolism.^68–70^ Gonadotropin-releasing hormone (GnRH) stimulates the secretion of luteinizing hormone (LH) and follicle-stimulating hormone (FSH), which in turn promote estrogen and androgen production. Estrogens enhance osteoblast activity, suppress osteoclastogenesis, and maintain trabecular architecture; estrogen deficiency after menopause is a major driver of osteoporosis.^71–73^ Kisspeptin neurons in the ARC are critical upstream regulators, integrating reproductive and metabolic signals with bone turnover.^74–76^

#### Hypothalamic-pituitary-thyroid (HPT) axis

The thyroid gland, under hypothalamic and pituitary control, is another important regulator of bone. Thyrotropin-releasing hormone (TRH) from the hypothalamus stimulates thyroid-stimulating hormone (TSH) release from the pituitary, which then induces thyroid hormone (TH) production.^77^ Physiological TH levels promote endochondral ossification, chondrocyte hypertrophy, and osteoblast maturation via Wnt/β-catenin and IGF-1 pathways.^78–81^ However, both hyperthyroidism and hypothyroidism disrupt bone homeostasis, leading to increased fracture risk.^82^

#### Other hypothalamic outputs

Beyond classical endocrine axes, hypothalamic neuropeptides also directly influence skeletal remodeling. Oxytocin (OXT), produced by the PVN and supraoptic nucleus (SON), stimulates osteoblast differentiation via BMP-2 and MAPK pathways, while also modulating osteoclast activity.^83^ Vasopressin (AVP) regulates osteoclast function by acting through specific receptors (Avpr1a, Avpr1b, Avpr2).^84,85^ Together, these hypothalamic outputs extend neural–endocrine control of bone beyond the canonical HPA, HPG, and HPT axes.

### Neuroendocrine and calcium-phosphorus metabolism

Calcium (Ca²⁺) and inorganic phosphate (Pi) are fundamental mineral constituents of bone, and their homeostasis is coordinately regulated by three key factors: PTH, 1,25-dihydroxyvitamin D, and the fibroblast growth factor 23 (FGF23)–α Klotho axis. These components interact through negative feedback loops to maintain systemic calcium and phosphate balance.^86–91^ Primary hyperparathyroidism, a common cause of reduced bone mineral density that predominantly affects postmenopausal women, illustrates the link between mineral homeostasis and the gonadal axis.^92,93^ This connection is further supported by evidence that GABAergic projections from the subfornical organ to the paraventricular nucleus modulate both PTH secretion and bone mass.^15^ Additionally, αKlotho stimulates growth hormone (GH) secretion from the pituitary gland via the ERK signaling pathway, thereby bridging mineral homeostasis with the growth hormone/IGF-1 axis.^94^ In summary, the calcium–phosphate metabolic network communicates extensively with the neuroendocrine system, enabling the organism to navigate various physiological and pathological challenges—such as growth, stress, and aging—while preserving skeletal homeostasis.^95–97^

### Circadian rhythm axis

Bone remodeling is tightly coupled to circadian rhythms orchestrated by the suprachiasmatic nucleus (SCN), the central biological clock. Light input through the retina is transmitted to the SCN, which synchronizes peripheral clocks across virtually all tissues, including bone.^98–100^ Disruption of circadian rhythms—for example, through chronic jet lag or night-shift work—impairs osteoblast differentiation, enhances osteoclast activity, and predisposes to skeletal fragility.^101^

At the molecular level, core clock genes such as *Per1/2*, *Cry1*, *Bmal1*, and *Clock* are expressed in osteoblasts and bone marrow stromal cells (BMSCs). The deletion of *Bmal1* promotes osteoblastogenesis and bone formation. Conversely, *Per1* deficiency inhibits bone growth and delays the mineralization process.^102–104^ Circadian regulation also intersects with neuroendocrine and autonomic pathways: SCN-driven sympathetic activity suppresses bone formation via *c-Myc* and *Cyclin-D* regulation, whereas neuromedin U links circadian cues with sympathetic modulation of skeletal remodeling.^105^

These findings highlight that bone is not only regulated by systemic hormones and neural inputs, but also follows intrinsic circadian oscillations that couple daily metabolic cycles with skeletal turnover.

## Classical peripheral nervous system regulation of bone

**Fig. 2:**
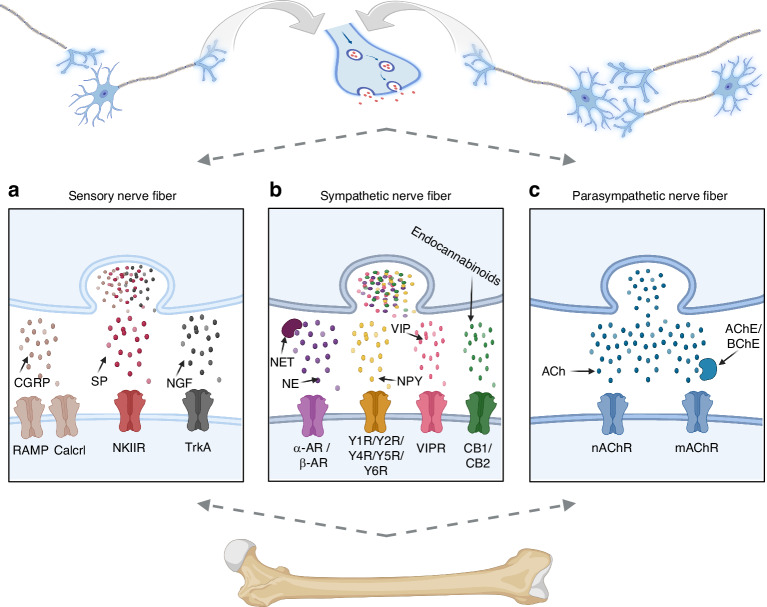
Key regulatory factors of bone mediated by the classical peripheral nervous system and its receptors. **a** In the peripheral sensory nervous system, calcitonin gene-related peptide (CGRP), substance P (SP), and nerve growth factor (NGF) function as key neurochemical mediators in the regulation of bone metabolism. They exert their effects by binding to specific receptors expressed in bone tissue, thereby modulating metabolic processes. **b** In the peripheral sympathetic nervous system, key neurotransmitters–including norepinephrine (NE), neuropeptide Y (NPY), vasoactive intestinal peptide (VIP), and endocannabinoids–regulate bone metabolism by binding to specific receptors in bone tissue. **c** In the peripheral parasympathetic nervous system, acetylcholine (ACh) regulates bone metabolism through its interaction with nicotinic (nAChR) and muscarinic (mAChR) receptors present in bone tissue. (Created with BioRender.com.) α-AR, alpha-adrenergic receptors; AChE, acetylcholineesterase; β-AR, beta-adrenergic receptor; BChE, butyrylcholinesterase; Calcrl, calcitonin receptor-like receptor; CB1, CB2, cannabinoid receptors type 1 and 2; NET, norepinephrine transporter; NK1R, neurokinin 1 receptor; RAMP, receptor activity-modifying protein; TrkA, tropomyosin receptor kinase A; VIPR, vasoactive intestinal peptide receptor; Y1R, Y2R, Y4R, Y5R, Y6R, neuropeptide Y receptor type 1, 2, 4, 5, and 6

The discovery of leptin as a regulator of bone remodeling shifted the field from merely documenting the presence of peripheral nerves in bone to mechanistic exploration of how the nervous system communicates with the skeletal microenvironment to govern bone homeostasis. Spatially, nerves are closely apposed to bone. The peripheral nervous system (PNS) bridges the central nervous system and the skeleton: afferents are collectively referred to as sensory nerves, whereas efferents include somatic motor nerves and autonomic nerves, the latter comprising the SNS and PSNS.^106^ This section focuses on the interactions and mechanisms linking peripheral sensory, sympathetic, and parasympathetic pathways to skeletal regulation (Fig. 2) (Table 2).

**Table 2:** Major peripheral nervous system neurotransmitters and their effects on OB, OC, and SSC

Cell types	Neural types	Neurotransmitters	Effects	Signaling pathway	References
OBs	Sensory nerves	CGRP	Promote	Calcitonin receptor-like receptor (CRL)	^119^
		SP	Promote	Wnt	^127^
		SEMA3A	Promote	Wnt	^140^
	Sympathetic nerves	NE	Promote	α1B-adrenergic receptor	^184^
			Inhibit	Β2-Adrenergic receptor, PKA-ATF4	^51,52^
	parasympathetic nervous	ACh	Promote/Inhibit	ACh receptors	^230,231^
OCs	Sensory nerves	CGRP	Inhibit	cAMP, NF-κB, OPG/RANKL	^118–121^
		SP	Promote	NF-κB, OPG/RANKL/RANK	^129,130^
		SEMA3A	Inhibit	Sema3A-Nrp1, ITAM、RhoA	^140^
	Sympathetic nerves	NE	Promote	α2-adrenergic receptors	^181,186^
			Promote	β-Adrenergic receptors, cAMP	^190–192^
	parasympathetic nervous	ACh	Inhibit	Nicotinic ACh receptors	^58^
BMSCs/SSCs	Sensory nerves	CGRP	Promote	MAPK(ERK1/2, p38), Hippo–YAP Wnt6/β-catenin, PKA/CREB/JUNB	^112–115,117^
		SP	Promote	–	^129^
		SEMA3A	Promote	Wnt/β-catenin/Nrp1	^141^
		SPP1	Promote	–	^34^
	Sympathetic nerves	FSTL1	Inhibit	BMP/TGF-β	^33^

*ACh* acetylcholine, *BMP* bone morphogenetic protein, *BMSCs* bone marrow mesenchymal stem cells, *cAMP* cyclic adenosine monophosphate, *CGRP* calcitonin gene-related peptide, *CREB* cAMP response element-binding protein, *FSTL1* follistatin-like 1, *ITAM* immunoreceptor tyrosine-based activation motif, *JUNB* transcription factor jun-B, MAPK mitogen-activated protein kinase, *NE* norepinephrine, *Nrp1* neuropilin-1, *OBs* osteoblasts, *OCs* osteoclasts, *OPG* osteoprotegerin, *PKA* protein kinase A, *RANK* receptor activator of nuclear factor-κB, *RANKL* receptor activator of nuclear factor-κB ligand, *RhoA* Ras homolog family member A, *SEMA3A* semaphorin 3A, *SP* substance P, *SPP1* secreted phosphoprotein 1, *TGF-β* transforming growth factor-beta

### Peripheral sensory axis

Sensory nerves innervate peripheral tissues to detect internal and external stimul.^19^ The cell bodies of primary sensory neurons reside in dorsal root ganglia (DRG) along the spinal cord and cranial nerve ganglia, and their axons project into bone to sense changes in bone density, mechanical loading, and metabolic activity, thereby modulating skeletal homeostasis.^107^ In humans, sensory fiber density is highest in periosteum, followed by marrow and cortex; fiber numbers decline with age without sex differences, forming a branching network throughout the periosteum.^108^

Pathologic fracture models highlight key roles for nerve growth factor-tropomyosin receptor kinase A (NGF–TrkA) signaling, Calcitonin gene-related peptide (CGRP), and substance P (SP)–positive sensory nerves in repair and remodeling; both sensory and autonomic inputs promote angiogenic ingrowth and sprouting, enhance osteogenic differentiation, and facilitate delivery of osteogenic factors to fracture sites.^109^ Collectively, sensory nerves modulate bone metabolism by releasing CGRP, SP, NGF, and semaphorin-3A (SEMA3A) within the bone microenvironment.^22,110^

#### Sensory neurotransmitters

##### Calcitonin gene–related peptide (CGRP)

CGRP exists as α- and β-isoforms: αCGRP derives from calcitonin-related polypeptide alpha (*CALCA*, alternative splicing of exon V), and βCGRP from calcitonin-related polypeptide beta (*CALCB)*. αCGRP predominates systemically, whereas βCGRP is mainly expressed in the enteric nervous system.^111^

CGRP^+^ sensory fibers are abundant in bone. αCGRP promotes osteogenesis via multiple routes: (1) direct stimulation of BMSC and osteoblast differentiation through the ERK1/2 and p38 MAPK pathways;^112^ (2) enhancement of BMSC migration and osteogenesis via the Hippo–YAP pathway,^113,114^ with additional evidence for p38 MAPK and Wnt6/β-catenin involvement.^115^ CGRP also inhibits BMSC adipogenic capacity,^116^ antagonizes osteoblast apoptosis, and—through CGRP receptor activation—improves tendon-to-bone healing.^117^ On the resorption side, CGRP maintains bone mass by suppressing osteoclastogenesis: it downregulates RANKL-induced NF-κB activation,^118,119^ shifts the OPG/RANKL balance in favor of anti-resorptive conditions,^120^ and directly inhibits human osteoclast formation via cyclic adenosine monophosphate (cAMP) signaling.^121^ Upon neuronal depolarization, presynaptic CGRP release augments osteogenic factor expression (e.g., BMP2, SP7) during regeneration; impaired CGRP release at fracture sites causes delayed union or non-union, whereas local CGRP supplementation enhances bone defect repair.^122–124^ Exogenous CGRP rescues nerve ablation–induced bone loss.^125^

Functioning as a key neuromodulatory signal, CGRP mediates cross-talk between the nervous and skeletal systems by enhancing osteoblast proliferation and differentiation, as well as inhibiting osteoclastogenesis and osteoclastic activity.

##### Substance P (SP)

SP acts via its receptor NK-1R to exert complex effects on bone homeostasis. SP deficiency (e.g., *Tac1*^*-/-*^ mice) features reduced bone formation with concomitant increases in resorption, leading to low bone mass, microarchitectural deterioration, and impaired biomechanics.^126,127^ In terms of bone formation, SP promotes proliferation and osteogenic differentiation of progenitors (e.g., BMSCs) through Wnt pathway activation.^128,129^ SP deficiency can accelerate early differentiation yet reduce the pool of metabolically active osteogenic cells, thereby lowering mineralization rates.^126^ In terms of bone resorption, SP shows context-dependent effects on osteoclasts: it can directly enhance NF-κB signaling in precursors, amplifying RANKL-driven osteoclastogenesis and resorptive activity.^130^ However, osteoclast recruitment may decline in *Tac1*-deficient mice, resulting in reduced resorption in some contexts.^126,131^ Following ovariectomy or sciatic neurectomy, mice demonstrated decreased skeletal SP levels, which were associated with subsequent bone loss; residual SP signaling remains essential to preserve bone integrity in this setting.^132,133^ In *S. aureus* osteomyelitis, SP/NK-1R aggravates bone damage by stimulating osteoclast-derived chemokines, driving neutrophil influx, and intensifying inflammation, while directly heightening osteoclast and neutrophil activity—together accelerating bone loss.^134,135^

Collectively, SP exerts a beneficial regulatory influence on the skeletal system. Under physiological conditions, it coordinates the coupling between bone resorption and bone formation to maintain bone homeostasis. In pathological states, however, SP tends to predominantly promote osteoclast activation, thereby contributing to pathological bone loss.

#### Sensory Guidance Cues Semaphorin-3A (SEMA3A)

During intramembranous ossification, SEMA3A expression increases in parallel with the spatial distribution of sensory fibers and precedes vascularization.^136^ Neuron-derived SEMA3A indirectly regulates bone remodeling by shaping sensory projections: neuron-specific deletion (*Sema3a*^synapsin−/−^ or *Nestin*^−/−^) reduces bone mass, mirroring the global knockout phenotype.^137^ DRG neurons promote osteogenesis by upregulating Cx43 and N-cadherin and activating Wnt signaling.^138^ Preclinical evidence suggests that sensory neuropathies associate with low bone mass and elevated fracture risk, underscoring the necessity of innervation for skeletal homeostasis.^139^ Mechanistically, SEMA3A binds a neuropilin-1 (NRP1)/plexinA1 receptor complex to trigger divergent downstream programs in osteoclasts vs osteoblasts. In stromal cells, SEMA3A–NRP1 stimulates canonical Wnt/β-catenin signaling, thereby promoting osteogenesis.^140^ Sensory-derived SEMA3A further reinforces BMSC osteogenesis via a Wnt/β-catenin/NRP1 positive-feedback loop.^141^ In osteoclasts, SEMA3A–NRP1 inhibits immunoreceptor tyrosine-based activation motif (ITAM) and ras homolog family member A (RhoA) signaling to block RANKL-driven differentiation,^142^ and NRP1 competes with the triggering receptor expressed on myeloid cells 2 (TREM2) –DNAX-activating protein of 12 kD (DAP12) complex for PlexinA1 binding to further restrain osteoclastogenesis.^143^ In the setting of radiation injury, SEMA3A promotes apoptosis of osteoclast-lineage cells, suggesting that SEMA3A may oppose radiation-associated bone loss.^144^ Therapeutically, systemic SEMA3A increases bone mass, accelerates regeneration, and improves vascularization and biomechanical strength of repair tissue;^140,145^ it also prevents bone loss in menopausal mouse models.^143^

Thus SEMA3A offers a “triad” mechanism—anti-bone resorption, pro-bone formation, and neural modulation—as a promising target in skeletal disease.

#### Additional sensory modulators

##### Prostaglandin E2 Receptor Subtype 4 (EP4)

The PGE2–EP4 pathway is central to bone metabolism, participating in homeostasis, load responsiveness, and regeneration. Osteoclast precursors downregulate EP2/EP4 during differentiation, potentially evading PGE2-mediated anti-resorptive effects.^146^ PGE2 selectively activates EP4 (not other EP subtypes) to bidirectionally tune resorption and formation; EP4 agonists restore trabecular bone volume in ovariectomy (OVX) or immobilized rats, enhance fracture callus formation, and increase osteoid and osteoblast numbers.^147^ EP4 deficiency reduces bone mass in aged male mice and impairs fracture healing.^148^ With mechanical loading, cyclooxygenase-2 (COX-2) loss in osteoblasts or EP4 deletion in sensory neurons diminishes load-induced formation; TrkA deletion in sensory neurons similarly suppresses this process, indicating a neuro–bone coupling through this pathway.^20^ Mechanistically, osteoblast-derived PGE2 activates EP4 on sensory neurons, drives hypothalamic CREB phosphorylation, suppresses sympathetic activity, and thereby promotes bone formation.^19,23^

In summary, EP4 primarily exerts anabolic effects on osteoblasts by promoting differentiation and bone formation. Its actions on osteoclasts are more multifaceted: while it indirectly regulates osteoclastogenesis via osteoblast-mediated pathways, it can also directly modulate osteoclast function under pathological conditions, thereby contributing to disease progression.

##### Tropomyosin Receptor Kinase A (TrkA)

The NGF–TrkA axis orchestrates sensory innervation, skeletal development, mechanoadaptation, and repair. The binding of nerve growth factor (NGF) to its receptor TrkA activates the ERK-Bcl2 pathway, thereby enhancing the regenerative capacity of BMSCs. This signaling cascade upregulates the expression of transforming growth factor-beta (TGF-β) and bone morphogenetic protein 9 (BMP-9), and promotes the phosphorylation of Smad and p38 MAPK, which in turn potentiates BMSC differentiation.^149–151^ During early skeletal development, expansion of chondro-osseous progenitors requires dense innervation by NGF-responsive TrkA^+^ sensory fibers.^152^ In embryonic femur, TrkA^+^ axons align along the periosteal surface, spatially overlapping with NGF near ossification centers; selective inactivation of TrkA (e.g., *TrkA*^F592A^ mice) impairs innervation and delays vascularization of primary/secondary ossification centers, reduces Osx^+^ osteoprogenitors, and causes femoral growth retardation—supporting NGF as an essential neurotrophic factor for normal ossification via TrkA.^153^ In mature bone, NGF–TrkA is an early mechanotransduction signal and is required for maximal load-induced formation.^154^ Osteoblast-derived NGF engages TrkA on resident sensory fibers after loading to support osteogenesis.^155^ Context matters: in soft-tissue injury, NGF-driven overgrowth of TrkA^+^ axons fuels aberrant endochondral differentiation, suggesting therapeutic value of pathway inhibition.^156^ Conversely, in fracture repair, NGF/TrkA expression peaks during chondrogenesis; local β-NGF injection improves healing (less cartilage, greater bone volume, higher trabecular number/connectivity and mineral density),^157^ and TrkA agonists (e.g., gambogic amide) enhance mechanoadaptation.^158^

Overall, TrkA plays a crucial role in promoting bone formation, fracture healing, skeletal development, and adaptive remodeling. Additionally, it helps maintain bone resorption homeostasis by inhibiting osteoclast generation.

##### Transient Receptor Potential Vanilloid 1 (TRPV1)

The polymodal receptor TRPV1, a nonselective cation channel, is highly expressed in sensory neurons and crucial in bone homestasis. TRPV1 activation induces DRG release of CGRP, forming a TRPV1–CGRP neuro-bone axis that boosts osteoblast proliferation, migration, and differentiation, accelerating bone defect repair via Hippo–YAP modulation (LATS1/MOB1 phosphorylation, YAP nuclear translocation).^159^ Neuronal *Trpv1* knockdown/knockout impairs osteogenesis, which can be rescued by exogenous CGRP.^159,160^ TRPV1 loss directly restrains osteoclastogenesis by reducing Ca^2+^ oscillation frequency/amplitude, NFATc1 nuclear translocation, and expression of DC-STAMP and cathepsin K, while also dampening osteogenesis.^160^ In osteoporosis, TRPV1 ablation or inhibition restores osteoclast homeostasis and prevents OVX-induced bone loss.^161^ TRPV1 contributes to bone pain: acidic microenvironments (tumor or osteoclast-derived protons) activate TRPV1/ASIC3 in bone-afferent neurons, driving myeloma-related pain.^162^ Both Aδ and C bone afferents express TRPV1, whose agonists sensitize neurons and exacerbate pathologic pain.^163^ OVX elevates TRPV1 + DRG neurons and CGRP expression with mechanical allodynia; TRPV1 inhibition alleviates pain and improves bone metabolism.^164^ Deinnervated osteoporotic femurs show a higher proportion of TRPV1-immunoreactive neurons, implicating TRPV1 in pathologic osteo-sensory signaling.^165^ Taken together, TRPV1 maintains bone homeostasis through dual regulation: suppressing bone resorption and enhancing bone formation.

### Peripheral sympathetic axis

#### Sympathetic nerve fibers

A key vertebrate innovation, the SNS, arises from neural crest–derived progenitors.^166^ It comprises noradrenergic fibers (primary transmitter norepinephrine, NE) and cholinergic fibers.

##### Noradrenergic sympathetic fibers

Circulating norepinephrine originates largely from peripheral sympathetic terminals.^167^ Sympathetic fibers commonly express tyrosine hydroxylase (TH), the rate-limiting enzyme converting L-tyrosine to L-DOPA. In bone, TH^+^ fibers reside in marrow, periosteum, and entheses; innervation is rich in the metaphysis/diaphysis but sparse in the epiphysis.^168,169^ The SNS shapes the bone/bone-marrow niche, and NE influences proliferation or apoptosis across multiple cell types.^170,171^ At the molecular level, catecholamines elevate intracellular cAMP and can potentiate BMP-induced osteoblast differentiation and bone formation.^172^ In osteoblasts, sympathetic signaling suppresses CREB phosphorylation (reducing proliferation) while enhancing activating transcription factor 4 (ATF4) phosphorylation and upregulating RANKL to stimulate osteoclastogenesis.^173^ CREB is itself a positive regulator of osteogenesis; expression of CREB-linked genes (*Cyclin D1/D2*, *Cyclin E1*, *Per1*, *c-Myc*) is altered in *Creb*-deficient models.^173,174^ In humans, higher sympathetic activity correlates inversely with trabecular microarchitecture, compressive strength, serum procollagen type I N-terminal propeptide (PINP), and plasma osteopontin, echoing murine data that sympathetic overactivity suppresses bone formation and degrades trabecular structure.^175^

##### Adrenergic sympathetic fibers

While noradrenergic fibers typically lower bone mass, the skeletal role of sympathetic cholinergic fibers long remained unclear. These fibers contact osteocytes through a GFRα2–neurturin (NRTN) trophic mechanism; osteocytes require cholinergic innervation for survival and network connectivity.^176^ Loss of skeletal cholinergic fibers causes osteocyte atrophy and bone loss from reduced formation, whereas exercise expands cholinergic sympathetic fibers via IL-6–dependent mechanisms to strengthen trabeculae.^176^ Systemic NRTN increases bone mass in OVX mice, implicating cholinergic sympathetic fibers in bone accrual via NRTN.^177^

#### Sympathetic adrenergic receptors

NE signals through α-adrenergic (α-AR) and β-adrenergic (β-AR) receptors. Osteoblasts and osteoclasts express multiple α/β subtypes, and receptor activation generally suppresses formation while promoting osteoclastogenesis, leading to bone loss.^178^

##### α-Adrenergic receptors (α-AR)

The α-adrenergic receptors play a significant role in regulating bone metabolism. Studies have shown that mRNAs encoding the α1A, α1B, and α1D subtypes are expressed in both bone marrow progenitor cells and osteoblasts, with α-adrenergic receptor mRNAs being commonly detected in human osteoblasts.^179–183^ Furthermore, α1B-adrenergic receptor signaling has been shown to promote osteoblast proliferation and bone formation by upregulating CCAAT/enhancer-binding protein δ (C/EBPδ).^184^ The α2-adrenergic receptors (α2-ARs), particularly the α2A and α2C subtypes present in bone cells, appear to suppress bone mass. This is supported by the high-bone-mass phenotype observed in female mice lacking both α2A- and α2C-ARs (α2A/α2C-ARKO), which results from elevated sympathetic tone and manifests as coupled increases in bone formation and decreases in resorption. The pro-osteoclastic role of α2-AR signaling was further confirmed by its capacity to promote osteoclast differentiation in vitro.^181,185,186^

In summary, α-adrenergic receptors play a critical role in bone metabolism by directly stimulating osteoblast activity to enhance bone formation, while also exerting indirect regulatory effects on osteoclasts.

##### β-Adrenergic receptors (β-AR)

The SNS regulates skeletal balance largely via β-ARs. Dopamine beta-hydroxylase (Dbh) deficiency (reduced catecholamine synthesis) yields late-onset high bone mass; the nonselective β-blocker propranolol increases bone in mice/rats, whereas β-agonists (isoproterenol) or β2-selective agonists (clenbuterol, salbutamol) cause low bone mass. Taken together, these findings support β-AR signaling generally suppressing bone formation.^55,187,188^ β1-AR promote bone anabolism : (1) β1-AR signaling induces chond rocyte exosomal miR-125 to enhance subchondral osteogenesis;^189^ (2) stress-induced β1/β2 activation drives osteoblastic miR-21 transcription and exosomal transfer to osteoclast precursors to promote osteoclastogenesis.^190^ β2-AR promotes bone catabolism: (1) β2-AR simultaneously suppresses bone formation and stimulates resorption via cAMP/PKA-driven RANKL upregulation;^191,192^ (2) osteocyte-specific β2-AR deletion reduces resorption;^52^ (3) isoproterenol elevates osteopontin (OPN) via β2-AR/cAMP to drive bone loss;^193^ (4) osteocytic β2-AR modulates RANKL/OPG and neuropeptides to regulate osteoclastogenesis;^194^ (5) glucocorticoids increase osteoblastic β2-AR expression/activity to exacerbate catabolism.^195^ Of note, β2-AR can in some contexts promote bone repair: NE–ADRB2 stimulates periosteal VEGFA and αCGRP, and via β2-AR/CREB/PFKFB3 promotes type-H angiogenesis and osteogenesis coupling.^196,197^

In summary, β1 and β2 exert opposing influences: β1 dominates mechanical anabolism, whereas β2 drives age-linked resorption.^198^

#### Additional sympathetic modulators

##### Norepinephrine transporter (NET)

Synaptic NE is cleared primarily by the presynaptic NET, which mediates 80%–90% of NE reuptake, limiting signal duration and replenishing stores.^199^ Osteoblasts and osteocytes also take up—but do not synthesize—NE via NET. Net-deficient mice lack NE reuptake, show reduced bone formation and increased bone resorption with low sympathetic outflow but elevated plasma NE, culminating in lower peak bone mass and strength.^200^ Differentiated osteoblasts display consistent NET uptake in vitro, and in vivo NET-specific NE uptake occurs in cortical bone lacunae.^199^ Together these data indicate: (1) NET is expressed in adult cortical bone cells; (2) adult cortex is a site of NET-mediated NE uptake; (3) sympathetic fibers populate the endosteal envelope; (4) NET expression/function declines with age. Interestingly, systemic pharmacologic NET inhibition did not impair early/mid-stage femoral healing and may improve late outcomes in mice, leaving NET’s role in regeneration nuanced.^201^

Taken together, NET serves as a neural checkpoint regulating bone metabolism. Reduced NET activity leads to amplified sympathetic tone, consequently shifting the bone equilibrium toward resorption and bone loss.

##### Endocannabinoid system (CB)

Constituents of the endocannabinoid system are expressed in bone,^202,203^ where endocannabinoids mediate their effects through binding to and activating the G protein-coupled receptors CB1 and CB2. Animal models show a dominant role in remodeling: CB1 loss increases bone mass and protects against OVX-induced loss.^204^ Neuron-specific CB1 deletion elevates bone mass with higher formation and lower osteoclastogenesis, consistent with a model in which CB1 activation on sympathetic terminals suppresses NE release, thereby modulating SNS output to bone.^205–208^ CB2 on osteoblasts/osteoclasts regulates proliferation and function; CB2 activation stimulates formation and suppresses resorption, whereas CB2 deficiency accelerates age-related bone loss.^209,210^ Human genetics supports clinical relevance: Significant associations have been identified between polymorphisms in the CNR2 gene (encoding the CB2 receptor) and both bone mineral density (BMD) and osteoporosis risk, with the most pronounced effects observed in women.^211^ Both CB1/CB2 antagonists can induce osteoclast apoptosis and inhibit survival factors.^204^ However, no studies have yet established the therapeutic efficacy of CB1/CB2 ligands in bone healing/regeneration,^212^ making this an open question.

Activation of CB1 primarily inhibits bone formation, whereas CB2 activation promotes bone formation and suppresses bone resorption, demonstrating opposing effects between the two receptors.

##### Neuropeptide Y (NPY)

NPY is a conserved peptide often co-released with NE. scRNA-seq of sympathetic ganglia shows that ~40% of neuronal clusters express high levels of NPY.^213^ Peripherally, NPY colocalizes with NE at sympathetic terminals and is co-released upon activation. High NPY associates with GC-induced trabecular deterioration and marrow adiposity; NPY-deficient mice exhibit high bone mass with robust trabecular and cortical microarchitecture and are protected from GC-induced skeletal defects and marrow adiposity.^214^ Osteocytic NPY loss also yields high-bone-mass and mitigates aging- and OVX-associated osteo-adipogenic imbalance.^215^ NPY acts through Y1R, Y2R, Y4R, Y5R, Y6R.^214,216^ Y1R is expressed throughout the osteoblastic lineage and in osteocytes;^217^ sympathetic inputs can reduce bone formation via Y1R on osteoblasts.^218^ Exogenous NPY reduces subchondral trabecular formation and microarchitecture in OVX rats and upregulates pyroptosis markers, whereas Y1R antagonists reverse these changes.^219,220^ Trabecular bone density and the rate of bone mineralization and formation are increased in Y2 knockout mice; effects are not humoral but likely central, as hypothalamic Y2R modulates autonomic outflow to bone.^55,221^ Centrally and peripherally, Y2R suppresses NE and corticosterone production/release, protecting bone under chronic stress.^222^

These findings indicate that NPY acts as a negative regulator of bone mass by suppressing the activity of both BMSCs and osteoblasts in the peripheral context.

##### Vasoactive intestinal peptide (VIP)

Classically linked to sympathetic pathways in bone, VIP-immunoreactive periosteal fibers disappear ipsilateral to thoracic sympathetic chain ganglionectomy (but not after dorsal root ganglionectomy), indicating sympathetic VIPergic innervation of periosteum and bone.^223^ VIP promotes osteogenesis: Surgical sympathectomy in mice induces a reduction in cancellous bone quality, downregulates VIP expression, and consequently impairs fracture healing.^224^ VIP also reduces the RANKL/OPG ratio in human BMSC-osteogenic cultures, suggesting direct cross-talk from osteoblasts to osteoclasts.^225^ VIP suppresses osteoclast differentiation by downregulating/attenuating NFATc1.^226^

In summary, VIP enhances bone formation by promoting osteoblast differentiation and inhibiting osteoclast activation.

### Peripheral parasympathetic axis

The parasympathetic nervous system, a division of the autonomic nervous system, is derived from neural crest cells and comprises neurons typically situated within or near target organs in the cranial, thoracic, and abdominal regions. It often exerts antagonistic effects on the SNS at numerous effector sites.^227,228^

#### Parasympathetic neurotransmitter

The PSNS terminals primarily release acetylcholine (ACh), acetylcholine is synthesized under the catalysis of choline acetyltransferase, stored in synaptic vesicles, and subsequently acts on nicotinic or muscarinic receptors, respectively.^229^ Within the skeletal system, osteoblasts express specific ACh receptors and essential cholinergic components, forming a BMP-2-regulated system that modulates bone remodeling and osteoblast proliferation and differentiation.^230–233^ Furthermore, systemic administration of the central cholinergic agonist donepezil downregulates sympathetic tone, upregulates parasympathetic activity, alters whole-body energy metabolism, and ultimately promotes bone accrual.^234,235^ Overall, the Ach system has been shown to promote osteoblast proliferation and induce osteoblast apoptosis.

#### Parasympathetic receptors

##### Nicotinic acetylcholine receptors (nAChRs)

Derived from the PSNS, ACh primarily acts on nicotinic acetylcholine receptors (nAChRs) expressed by osteoclasts. Activation of nAChRs by agonists promotes osteoclast apoptosis and attenuates bone resorption.^58,61^ Evidence from *α2 nAChR* subunit knockout mice demonstrates increased resorptive activity and reduced bone mass, identifying α2 as a positive regulator of bone accumulation.^58^ In contrast, epidemiological studies associate nicotine—an nAChR ligand—with lower bone mineral density and elevated fracture risk, implicating the *α7 nAChR* subtype.^236,237^ Mechanistically, prenatal nicotine exposure induces osteopenia in male offspring, while *α7 nAChR*-knockout mice exhibit impaired RANKL-induced osteoclastogenesis and consequently higher bone mass.^238–240^ Further studies reveal that nicotine elevates serum RANKL levels in wild-type mice and suppresses osteoclast function by inhibiting cathepsin K, MMP-9, and actin ring formation.^237,241^ Thus, α7-mediated vagal signaling serves as a critical modulator of osteoclast activity and bone homeostasis. Additionally, α7 nAChR is enriched in newly formed endochondral matrices and contributes to early osteogenic responses to mechanical stimulation^242,243^ Altered bone strength, microarchitecture, and turnover markers in *α9 nAChR*-deficient mice suggest a role for α9-containing receptors in skeletal maintenance.^244^

##### Muscarinic acetylcholine receptors (mAChRs)

M3 muscarinic acetylcholine receptor -mediated PSNS signaling acts as a positive regulator of bone mass—enhancing formation and inhibiting resorption.^62^ M3 activation improves trabecular microarchitecture, increasing bending stiffness and stimulating matrix synthesis.^245^ Moreover, M5 muscarinic acetylcholine receptor expression is also reduced in osteoporotic osteoblasts.^246^ However, the role of other types of muscarinic receptors in regulating bone metabolism remains unclear, and this represents an area that remains to be explored.

#### Additional parasympathetic factors

Acetylcholinesterase (AChE) and butyrylcholinesterase (BChE) are two key enzymes responsible for hydrolyzing and degrading acetylcholine (ACh), thereby terminating the transmission of nerve impulses.^247^ In addition to their roles in the nervous system, both enzymes are involved in the regulation of the skeletal system. Evidence supporting the AChE in bone metabolism demonstrates that *AChE*-knockout mice exhibit impaired trabecular and cortical bone microarchitecture compared to WT mice, and that administration of AChE inhibitors promotes bone formation.^248–250^ Notably, the role of the other cholinesterase, BChE, appears to be distinct: in adult mice, *BChE*-knockout results in enhanced trabecular and cortical bone microarchitecture relative to WT controls.^251^ In summary, AchE suppresses osteogenesis, whereas BchE enhances bone formation.

## “Yin-Yang” sympathetic-sensory control of the skeleton

In 2013, T. Fukuda and colleagues reported that the skeletal phenotype of global *Sema3A* knockout mice is attributable to SEMA3A-mediated sensory innervation, rather than an osteoblast-intrinsic role of SEMA3A, thereby advancing the concept that neurons regulate bone metabolism.^137^ Yet whether—and how—neurons control cell populations upstream of osteoblasts has remained incompletely understood. SSCs, recently delineated as the cells sitting at the apex of the osteogenic hierarchy, meet the “gold-standard” criteria for stemness (self-renewal and multipotency), exhibit shared surface markers, and play indispensable roles in bone homeostasis and repair. SSCs have thus emerged as promising therapeutic targets for skeletal regeneration.^28,252–254^

Innervation patterning has become a focal theme in stem-cell niche biology: both sympathetic and sensory nerve subtypes are now recognized as core elements of specific tissue niches. However, the cellular constituents and logic of the SSC niche remain only partly resolved.^255–262^ We therefore posit a working hypothesis: analogous to the homeostatic coupling of osteoblastogenesis and osteoclastogenesis, sensory versus sympathetic innervation subtypes may impose an opposing, “yin–yang” mode of control over SSC fate during development and regeneration. In this view, the spatial distribution and modal identity of nerve fibers would help set SSC lineage choices and functional states. Recent work has begun to substantiate this framework.

Our recent studies demonstrate that the loss of SLIT2, a canonical axonal chemorepellent protein, augments sympathetic innervation in bone, revealing sympathetic fibers as a negative regulatory component of the SSC niche. During growth, higher sympathetic density correlates inversely with SSC abundance, whereas pharmacologic or surgical sympathectomy markedly expands SSCs in vivo. Mechanistically, neuronal SLIT2 controls secretion of FSTL1 (follistatin-like protein-1) from sympathetic neurons, which then autonomously suppresses SSC self-renewal and osteogenic potential in vitro and in vivo. These data establish a neuronal SLIT2–FSTL1 axis as a previously unrecognized niche module for SSC regulation and provide functional evidence that sympathetic innervation is present and physiologically consequential within the SSC niche.^33^

In contrast to sympathetic fibers, work on mice lacking the p75 neurotrophin receptor (p75NTR), an essential neurotrophin signaling component, reveals a reduction in sensory innervation of bone and identifies sensory neurons as a positive regulatory element within the SSC niche. Indeed, chemoneurolysis of sensory fibers diminishes SSC numbers. Mechanistic studies show that p75NTR in sensory neurons controls SPP1 (osteopontin) expression, which promotes SSC self-renewal and osteogenic differentiation. Notably, p75NTR downregulation in dorsal root ganglion neurons is also observed in Alzheimer’s disease models, coinciding with sensory deficits and SSC loss across physiological and pathological conditions. These findings suggest that targeted remodeling of the neurogenic component of the SSC niche in Alzheimer’s disease (AD) could ameliorate neurogenic osteoporosis and accelerate fracture healing. Together, they establish the presence and physiological importance of sensory neurons within the SSC niche.^34^

**Fig. 3:**
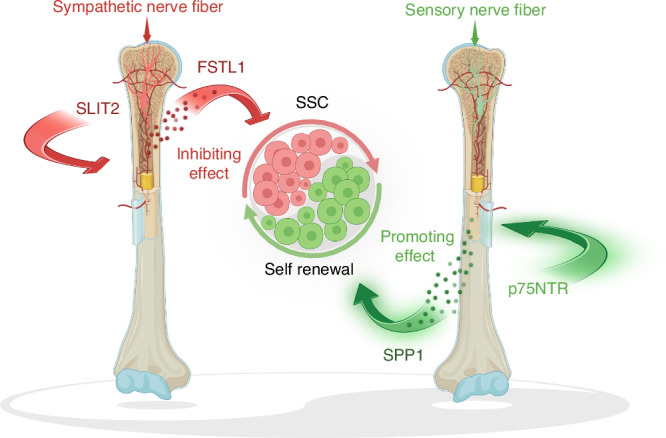
A novel "Yin-Yang" balance in sympathetic-sensory modulation of the skeleton. The SLIT2-FSTL1 axis, mediated by sympathetic nerve-innervated neurons, functions as a negative regulatory component within the skeletal stem cell (SSC) niche, thereby inhibiting SSC self-renewal. In contrast, the p75NTR-SPP1 axis, associated with sensory nerve-distributed neurons, acts as a positive regulator that promotes SSC self-renewal. Thus, sympathetic and sensory innervation serve as opposing (“yin-yang”) drivers that collectively define the SSC niche and guide SSC fate decisions. (Created with BioRender.com.)

Taken together, sympathetic and sensory innervation represent opposing (“yin–yang”) drivers that co-define the SSC niche and steer SSC fate decisions, thereby shaping bone formation during development, homeostasis, and repair (Fig. 3). This framework extends neuro-skeletal biology from direct actions on osteoblasts/osteoclasts to upstream stem-cell control, broadens the scope of skeletal regenerative medicine, and lays conceptual groundwork for diagnosing and treating neuro-associated osteogenic disorders.^32,263^

## Conclusions

This review synthesizes recent advances in neuro-skeletal interactions, focusing on established regulatory networks within the central nervous system and peripheral neural pathways responsible for bone innervation. It further expands the discussion to the emerging paradigm of local regulation mediated by the SSC neuro-microenvironment. The field’s focus has shifted from initial studies on the direct effects of neurons on osteoblasts and osteoclasts toward investigating upstream progenitor states, particularly SSCs, which are the ultimate orchestrators of bone remodeling and repair. We highlight the modality-specific roles of sensory versus sympathetic innervation as a “yin-yang” pair within the SSC niche, and outline their translational promise.

**Fig. 4:**
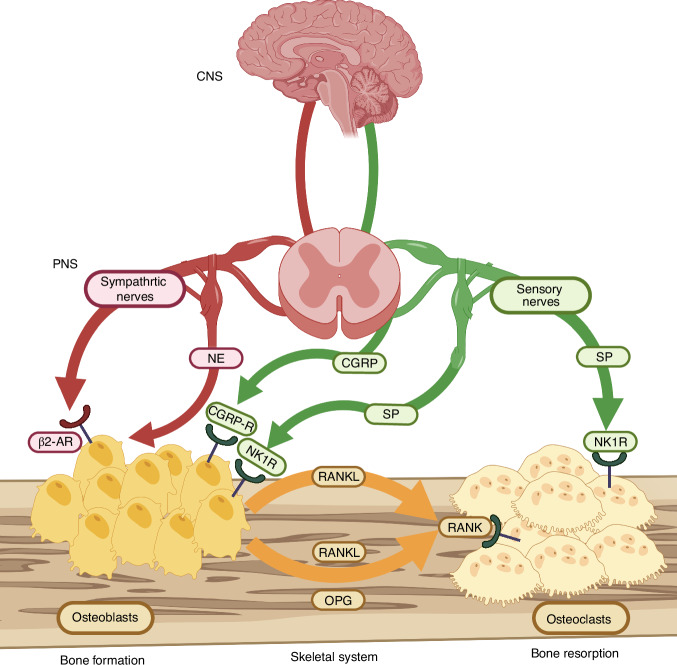
Key mediators and receptors of the classic regulatory axis linking the CNS, PNS, and skeletal system. Schematic diagram illustrating the coherent coupling framework of the CNS-PNS-skeletal system. This framework is mainly connected by sympathetic nerves and sensory nerves. In the classical neuro-osseous coupling framework, the central nervous system is connected to the peripheral nervous system through neural pathways, and classical coupling factors (such as NE, CGRP, and SP) are released to regulate the activity of osteoclasts and osteoblasts, thereby maintaining bone homeostasis. (Created with BioRender.com.) β-AR beta-adrenergic receptor, CGRP calcitonin gene-related peptide, CRGP-R calcitonin gene-related peptide receptor, CNS central nervous system, NK1R neurokinin-1 receptor, OPG osteoprotegerin, PNS peripheral nervous system, RANK receptor activator of nuclear factor-κB, RANKL receptor activator of nuclear factor-κB ligand, SEMA3A semaphorin-3A, SP substance P

Within the classical neuro-skeletal framework, the CNS integrates mechanical load signals conveyed by sensory nerves and metabolic signals from humoral fluids. After processing by specific nuclei, this integrated information primarily regulates bone metabolism by modulating central sympathetic outflow. NE released from sympathetic nerve terminals acts on the Adrb2 receptor of osteoblasts, inhibiting osteoblast proliferation by suppressing CREB phosphorylation, while promoting ATF4 phosphorylation to upregulate RANKL expression, thereby indirectly promoting osteoclastogenesis. In addition, sympathetic nerve terminals also release NPY and VIP, which constitute a complex neurotransmitter system for sympathetic nerves to regulate bone metabolism. Complementing this negative sympathetic regulation is the direct pro-osteogenic effect of sensory nerves. Beyond transmitting signals to the CNS via the PGE2-EP4 signaling pathway, sensory nerve terminals themselves release CGRP, SP, and SEMA3A into the bone microenvironment, which act directly on OBs, BMSCs, and OCs, triggering downstream signaling pathways conducive to bone formation. These neurotransmitters serve as direct mediators of communication between neural signals and skeletal cell responses, thereby enabling neural regulatory signals to engage canonical coupling pathways, such as the RANKL/OPG axis, to achieve coordinated integration of bone resorption and formation. Furthermore, the endocrine, metabolic, and circadian axes operate through hormones that enter bone tissue via the circulation or by driving oscillatory expression of local clock genes in the skeleton. These axes work in concert with the neural-skeletal loop through complex interactive networks to maintain systemic bone homeostasis under diverse physiological conditions (Fig. 4).

Within the framework of neural regulation of skeletal homeostasis, a central clinical and scientific challenge lies in balancing the dual, often opposing, roles of the nervous system in pain and bone repair. Following fracture, NGF-mediated pathological nerve sprouting represents a key driver of pain.^264–266^ In osteoarthritis, aberrant osteoclast activation promotes sensory axon growth in subchondral bone via Netrin-1 secretion, thereby contributing to pain pathways.^267,268^ Conversely, the fracture repair process itself requires spatiotemporally controlled sensory nerve ingrowth, which is crucial for initiating and promoting bone regeneration.^152,266,269^ In the osteoporotic fracture microenvironment, specific inflammatory osteoclasts secrete SEMA3A, impairing bone repair by inhibiting axonal regeneration of CGRP^+^ sensory neurons.^270^ Collectively, these findings suggest that an ideal therapeutic strategy should aim for spatiotemporally precise modulation of nerve distribution and function, rather than broad suppression. For instance, a combined strategy that inhibits pathological nerve sprouting while locally delivering pro-healing neurotrophic factors (e.g., SPP1 derived from sensory neurons) may achieve the dual benefit of analgesia and enhanced bone repair. Age-related neural remodeling—characterized by sympathetic overactivation and diminished sensory innervation—may at least partially explain the concurrent acceleration of bone loss and impairment of fracture healing in the elderly.^271–273^ Therefore, future translational research should extend beyond traditional strategies focused on single “bone targets” and instead focus on restoring and maintaining the integrated homeostasis of the “neuro-skeletal axis.” Translational strategies may evolve along two complementary paths: first, drug repurposing—such as exploring β-adrenergic receptor blockers (e.g., propranolol) to suppress excessive sympathetic activity and ameliorate age-related bone disorders; and second, developing novel neural-targeting therapies—for example, designing biologic agents that mimic the pro-regenerative signaling of SPP1 or antagonize the inhibitory function of SEMA3A. These approaches seek to synergistically promote fracture healing while effectively managing pain, ultimately paving the way for an integrated therapeutic paradigm that concurrently improves bone strength and repair capacity.

Although existing studies have established a theoretical foundation, a significant gap remains in translating these findings into clinical applications: causal circuit maps linking defined neuronal subtypes to SSC states; cell-type-specific and temporally precise perturbations in vivo; spatial multi-omics of the nerve–SSC interface; and human validation and safety of neuromodulatory strategies. Addressing these challenges will convert the emerging blueprint of the neural–SSC axis into actionable interventions for skeletal disorders.
